# Prevalence of cryptosporidiosis in renal transplant recipients presenting with acute diarrhea at a single center in Pakistan

**DOI:** 10.12860/jnp.2014.25

**Published:** 2014-10-01

**Authors:** Kapeel Raja, Zaigham Abbas, Syed Mujahid Hassan, Nasir Hassan Luck, Tahir Aziz, Muhammed Mubarak

**Affiliations:** ^1^Department of Hepatogastroenterlogy, Sindh Institute of Urology and Transplantation, Karachi, Pakistan; ^2^Department of Nephrology, Sindh Institute of Urology and Transplantation, Karachi, Pakistan; ^3^Department of Pathology, Sindh Institute of Urology and Transplantation, Karachi, Pakistan

**Keywords:** Cryptosporidium, Diarrhea, Immunocompromised, Renal transplant patients, Stool analysis

## Abstract

*Background: * Cryptosporidium is an intracellular protozoan organism which causes diarrhea, both in immunocompetent and immunocompromised hosts. Renal transplant recipients are prone to develop a variety of infections including protozoal infections.

*Objectives: * The aim of this study was to determine the prevalence of cryptosporidiosis in our renal transplant recipients presenting with acute diarrhea.

*Patients and Methods:* During six months of the study, 644 renal transplant recipients presented with acute diarrhea. Single stool sample was obtained for detailed analysis including gross and microscopic examination for red blood cells, pus cells, ova, cysts, and protozoa. The modified Ziehl-Neelsen (ZN) staining was done to identify the oocysts of cryptosporidia.

*Results: * Out of 644 renal transplant patients, oocysts of cryptosporidia were identified in 343 patients (53%). Detailed stool analysis of these patients showed the presence of numerous pus cells in 27 (7.9%) patients, co-infection with *giardia intestinalis* cysts in 15 (4.3%), and *entamoeba histolytica* cysts in 10 (2.9%). In all, out of 343 patients, 43 (12.5%) had dual infection with bacteria and protozoa in addition to cryptosporidiosis.

*Conclusions: * Cryptosporidium is an important pathogen causing acute diarrhea in renal transplant recipients in our set up. Stool examination is usually negative for pus cells. It is recommended that in all transplant recipients presenting with acute diarrhea modified ZN staining should be done to rule out cryptosporidiosis in highly endemic areas like Pakistan.

**Implication for health policy/practice/research/medical education:**

Cryptosporidiosis can readily occur in renal transplant recipients and should be considered in the differential diagnosis of acute diarrhea in these patients. It is important that modified Ziehl-Neelsen staining of stools should be done in all such cases to identify the oocysts of cryptosporidium especially in highly endemic areas like Pakistan.

## 1. Background

Cryptosporidium is one of the intestinal protozoal organisms belonging to the subclass of coccidia (1). In 1895, Clarke described the oocysts of this parasite in the gastric epithelial cells of the mouse (1) and in 1976, the first human case was reported (2-4). It typically presents with voluminous watery diarrhea, abdominal pain, weight loss, dehydration, and electrolyte imbalance which can threaten graft survival in renal transplant patients (5). Its transmission occurs primarily by the fecal-oral route (6). In the immunocompetant individuals, the disease is often benign and self-limited. In immunocompromised hosts, patients with acquired immunodeficiency syndrome (AIDS), renal transplant recipients and in cancer patients, cryptosporidial infection can persist for prolonged periods and can lead to serious complications (7-9).

There are very few studies on the prevalence of cryptosporidial infection in Pakistan and none in renal transplant recipients from this part of the world (10-13). The few studies done in other settings show that the prevalence of cryptosporidiosis is quite high, approaching that reported from some other third world countries (10-13). However, as alluded to earlier, the situation in the renal transplant patients in this country is entirely unknown. This patient population represents one of the main targets of this zoonotic disease. A few studies done on this patient population in neighboring countries show that cryptosporidial infection is also frequent in this population (14).

## 2. Objectives

The aim of this study was to determine the prevalence of cryptosporidiosis in our renal transplant recipients presenting with acute diarrhea. We also sought secondarily to determine the possible risk factors predisposing to infection with cryptosporidium in this population.

## 3. Patients and Methods

### 3.1. Patients

A total of 644 renal transplant patients were admitted with acute diarrhea in our institute during a period of six months from September 2010 to March 2011. All patients received live related renal transplants from siblings, parents or spouses and were maintained on standard triple immunosuppressive regimen (14). The immunosuppressive drugs comprised of cyclosporine/FK506, azathioprine/mycophenolate mofetil (MMF), and steroids. Cyclosporine was started at a dose of 6 mg/kg body weight which was reduced to 4 mg/kg body weight by the end of six months. During initial month following transplantation, cyclosporine levels were maintained at C0 level of 250-350 ng/ml or C2 level of 1200-1700 ng/ml. The starting and maintenance dose of azathioprine was 1.5-2 mg/kg body weight. Steroids were started at a dose of 0.5 mg/kg body weight and gradually reduced to 7.5-10 mg/day by the end of three months.

Acute diarrhea was defined as 3 or more loose or watery stools per day of less than 14 days in duration (15). Single stool sample was taken from each patient in sterile plastic container and sent to the pathology department on first day of admission for microscopic examination for pus cells, ova, and cysts. Modified Ziehl-Neelsen (ZN) staining method was used to identify cryptosporidial oocysts (16,17). Pus cells were semiquantitatively assessed as numerous (≥10 cells/HPF) or few (<10 cells).

Patients’ demographic, clinical and laboratory data were recorded on a proforma for subsequent analysis. Patients were subdivided into two groups based on the results of stool examination for cryptosporidial oocysts; cryptosporidial positive and negative groups. These were compared with respect to demographic, clinical and some laboratory parameters to determine the possible risk factors for the cryptosporidial infection.

### 3.2. Ethical issues

Informed consent was obtained from all patients for participation in the study and the study was conducted in accordance with the principles laid down in the declaration of Helsinki.

### 3.3. Data analysis

Statistical analysis was carried out using IBM compatible statistical package for social sciences (SPSS) for Windows version 10.0 (SPSS, Chicago, IL, USA). Simple descriptive statistics such as mean ± standard deviation (SD) were used for continuous variables such as age and clinical and laboratory parameters. Numbers (percentages) were used for categorical data, such as the prevalence of cryptosporidial infection. Student’s t-test and Chi-square test were used as appropriate to compare the demographic, clinical and laboratory differences among the two groups. P value of 0.05 was considered statistically significant.

## 4. Results

**Figure 1:**
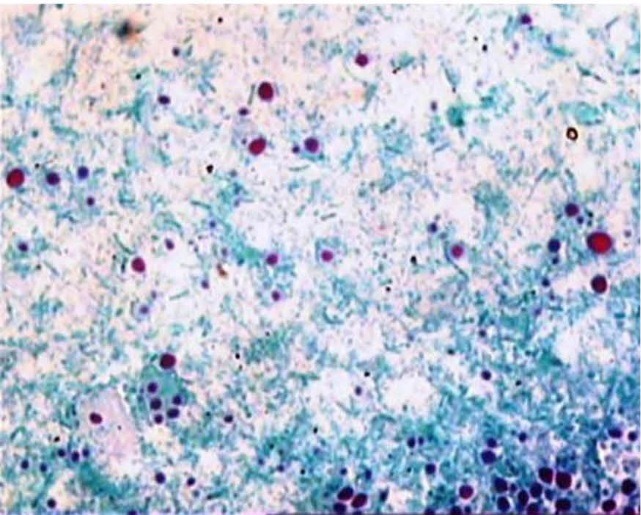
Smear shows numerous round to oval oocysts of cryptosporidium in a direct smear prepared from the stool sample from one of the study patients. (Modified Ziehl-Neelsen staining, ×1000).

The main demographic, clinical and laboratory parameters of the study patients are shown in Table 1. Out of 644 renal transplant patients who presented with acute diarrhea in the posttransplant OPD during the study period, oocysts of cryptosporidia were identified in 343 (53%) patients, in fairly large numbers in all cases (Figure 1). This constituted the cryptosporidium positive group. Of these, 213 (62%) were males and 130 (38%) females. Among cryptosporidium negative group, 169 (56%) were males and 132 (44%) were females. There was no significant difference in the gender distribution in the two groups. The mean age was 32.1±11 years in cryptosporidium positive group and 36±13 years in cryptosporidium negative group, with no statistically significant difference among the two groups. In addition to the presence of cryptosporidia, numerous pus cells (≥10 cells/HPF) were reported in the stool examination in 27 (7.8%) patients, co-infection with giardia intestinalis cysts in 15 (4.4%) and entamoeba histolytica cysts in 10 (2.6%) patients of the cryptosporidium positive group. The same parameters in the cryptosporidium negative group were found in 35 (11.6%), 9 (2.9%), and 14 (4.63%) respectively with no significant difference in the two groups. Overall, 43 (12.5%) patients of the cryptosporidium positive group were deemed to be suffering from co-infection as suggested by the presence of cysts of other organisms or superadded bacterial infection resulting in numerous pus cells in the stools. So these patients were treated with ciprofloxacin and metronidazole in addition to nitazoxanide for mixed infection and all responded favorably to the combined therapy.

**Table 1:** The main characteristics of study patients with acute diarrhea associated with cryptosporidiosis compared with those with no cryptosporidiosis.

**Parameters**	**Cryptosporidium positive** **group ( n= 343)**	**Cryptosporidium negative group ( n= 301)**	**p-value**
**Age**			
Mean± SD (years)	32.1±11	36 ±13	NS
**Gender,** n (%)			
Male, n (%)	213 (62%)	169 (56%)	
Female, n (%)	130 (38%)	132 (44%)	NS
**Duration of transplant**			
< 2 years	44 (12.8%)	36 (11.9%)	
2-5 years	151 (44%)	106 (35.1%)	
6-10 years	53 (15.4%)	46 (15.2%)	
11-15 years	78 (22.7%)	99 (32.7%)	
>15 years	17 (4.95%)	14 (4.9%)	NS
**Stool analysis**			
**Pus cells**			
Numerous	27 (7.87%)	35 (11.58%)	
Few	29 (8.45%)	21 (6.95%)	
Red Blood Cells	29 (8.45%)	27 (8.94%)	NS
**Co-infection**			
Giardia intestinalis	15(4.37%)	9 (2.9%)	
Ent. Histolytica	10 (2.9%)	14 (4.63%)	
Mixed infection	18 (5.24%)	17 (5.64%)	NS

## 5. Discussion

Cryptosporidium is a common intracellular protozoan pathogen which causes severe and life-threatening diarrhea in immunocompromised hosts (18). The infection has worldwide distribution. It is endemic in many tropical countries, including Pakistan. There are few epidemiological studies on the prevalence and incidence of this protozoal infection in this country. The few studies that are available in the literature show that the infection is quite common. However, there is no information on the prevalence of the infection in renal transplant patients from this part of the world.

This study pertains to a special subset of the population, that is, the prevalence of cryptosporidial infection in renal transplant patients in Pakistan. As the renal transplant recipients receive life long immunosuppressive drugs, they are not only prone to develop opportunistic infections but they get infected even with lower dose of organisms (19). In immunocompetant hosts, cryptosporidiosis is responsible for a self-limited disease lasting for 10–14 days (20). The prevalence in immunocompetant patients is higher in less developed countries (5 to 20 %) than in Europe (1–2 %) where transmission is linked to swimming-pool water, day-care centers and the use of communal restrooms (21-23). Infection with cryptosporidium is also a frequent cause of traveler’s diarrhea (6,21). Previous studies done in immunocompromised and renal transplant recipients showed the prevalence of cryptosporidiosis in India, Turkey, and Iran of 16.6%, 18.8%, and 11.5% respectively (6,7,19). In contrast, the prevalence of the organism in this study is very high. This may be mainly related to the environmental and poor hygienic conditions prevalent in most of the rural Pakistan. In addition, cryptosporidium has a potentially high infectivity, and ready transmission in immunocompromised patients is common.

Regarding the risk factors predisposing to the infection, none other than immunosuppression itself, was found significantly different in cryptosporidium positive and negative groups. It is possible that this is due to relatively small size of the study population. We also did not systematically analyze such factors as rural or urban origin of the patients, water sources used and household hygienic conditions in this study. As discussed in the beginning, the main aim of this study was to get the feel of the situation. A very high prevalence of cryptosporidiosis in this patient population needs to be studied further and for appropriate measures to be taken. For the proper management of cryptosporidiosis, source of spread of infection should be sought so that appropriate preventive measures such as boiling water for drinking, hand washing, changes in lifestyle and dietary habits may be undertaken. Other measures include hand washing after diaper changing, avoiding swallowing water while swimming, and avoiding contact with infected people or young pets (21). We did not systematically explore the sources of infection or spread of the organism in this population. However, we are planning to study the above aspects in future to identify the above aspects of the disease.

The current study has certain limitations too. It is a brief, cross-sectional survey of exploratory nature of a relatively specific section of the population. Only single fecal sample per patient was obtained and analyzed. No sample concentration technique was employed. Although the compliance of renal transplant patients is very high at our center, a selection bias in favor of more severe episodes of diarrhea can not be entirely excluded. In spite of the above limitations, the rate of cryptosporidial oocyst passage in the stools is very high. This calls for more studies on a larger scale to fully determine the true epidemiologic characteristics of the infection in the country. We think that our findings add new information to the existing gap in knowledge on this disease in the renal transplant population.

## 6. Conclusions

In conclusion, cryptosporidiosis can readily occur in renal transplant recipients and should be considered in the differential diagnosis of acute diarrhea in these patients. It is important that modified ZN staining of stools should be done in all such cases to identify the oocysts of cryptosporidium especially in highly endemic areas like Pakistan.

## Authors’ contributions

All authors wrote the paper equally.

## Conflict of interests

The authors declared no competing interests.

## Funding/Support

None.
